# *In silico* identification, characterization expression profile of WUSCHEL-Related Homeobox (WOX) gene family in two species of kiwifruit

**DOI:** 10.7717/peerj.12348

**Published:** 2021-10-28

**Authors:** Chen Feng, Shuaiyu Zou, Puxin Gao, Zupeng Wang

**Affiliations:** 1 Lushan Botanical Garden, Chinese Academy of Sciences, Jiujiang, China; 2 Innovative Academy of Seed Design, Chinese Academy of Sciences, Beijing, China; 3 Engineering Laboratory for Kiwifruit Industrial Technology, Chinese Academy of Sciences, Wuhan, China; 4 Key Laboratory of Plant Germplasm Enhancement and Specialty Agriculture, Wuhan Botanical Garden, Chinese Academy of Sciences, Wuhan, China

**Keywords:** *Actinidia chinensis*, *A. eriantha*, Whole-genome duplication, Expression profiles, Conserved motif

## Abstract

The WUSCHEL (WUS)-related homeobox (*WOX*) gene family is a class of plant-specific transcriptional factors and plays a crucial role in forming the shoot apical meristem and embryonic development, stem cell maintenance, and various other developmental processes. However, systematic identification and characterization of the kiwifruit *WOX* gene family have not been studied. This study identified 17 and 10 *WOX* genes in *A. chinensis* (Ac) and *A. eriantha* (Ae) genomes, respectively. Phylogenetic analysis classified kiwifruit *WOX* genes from two species into three clades. Analysis of phylogenetics, synteny patterns, and selection pressure inferred that *WOX* gene families in Ac and Ae had undergone different evolutionary patterns after whole-genome duplication (WGD) events, causing differences in *WOX* gene number and distribution. Ten conserved motifs were identified in the kiwifruit *WOX* genes, and motif architectures of *WOXs* belonging to different clades highly diverged. The *cis*-element analysis and expression profiles investigation indicated the functional differentiation of *WOX* genes and identified the potential *WOXs* in response to stresses. Our results provided insight into general characters, evolutionary patterns, and functional diversity of kiwifruit *WOXs*.

## Introduction

Homeobox (HB) proteins were primarily identified in *Drosophila*, and they belong to a large transcriptional factor (TF) family harboring a short stretch of amino acids (60–66 residues) conserved DNA-binding domain (referred to as homeodomain) (Mukherjee, Brocchieri & Bürglin, 2009; Holland, 2013; Alvarez et al., 2018). HB proteins could be found in all eukaryotic species tested (Liu et al., 2014b). The homeodomain (HD)-containing transcriptional factor family has been identified in both monocots and dicots (Ariel et al., 2007; Liu et al., 2014b). The HB superfamily can be classified into six subfamilies, including homeodomain-leucine (HD-Zip), plant homeodomain (PHD)-finger, BELL, zinc finger-homeodomain (ZF-HD), WUSCHEL (WUS)-related homeobox (WOX), and KNOTTED1-like-homeobox (KNOX) (Ariel et al., 2007; Mukherjee, Brocchieri & Bürglin, 2009; Liu et al., 2014b). Previous researches have verified that members of the *WOX* subfamily participate in many plant biological processes, such as the formation and maintenance of the shoot apical meristem (SAM) (Meng et al., 2019). The *WOX* subfamily specifically binds to the target region by the homeodomain to activate or depress the expression of the target gene in plants (Mukherjee, Brocchieri & Bürglin, 2009; Jha, Ochatt & Kumar, 2020; Tvorogova et al., 2021). Genome-wide identification of the *WOX* subfamily has been performed in several plants, including Arabidopsis, rice, maize, walnut, physic nut, grapes, peach, pear, apricot, coffee, and poplar (Li et al., 2020; Shafique Khan et al., 2021).

The *WOX* gene family has often been classified into three clades which include the ancient clade, the intermediate clade, and the modern/WUS clade (Tvorogova et al., 2021). Among the 15 *WOX* genes in Arabidopsis, *AtWOX10*, *AtWOX13*, and *AtWOX14* belong to the ancient clade (Tvorogova et al., 2021). The *AtWOX10* gene is a presumptive pseudogene owing to its undetectable gene expression in Arabidopsis plants (Deveaux et al., 2008). While *AtWOX13* and *AtWOX14* play central roles in regulating the development of flowers, fruits, and conductive tissues (Deveaux et al., 2008; Costanzo, Trehin & Vandenbussche, 2014; Jha, Ochatt & Kumar, 2020). However, WOX genes in the ancient clade expressed differently in different species, indicating the species-specific function of ancient clade WOX genes (Alvarez et al., 2018). The *AtWOX13* gene regulates fruit development, the number of lateral roots, and flower time (Deveaux et al., 2008; Romera-Branchat et al., 2013). The *AtWOX14* usually affects plant growth and the formation of conductive tissues, and the deletion of *AtWOX14* induces plant dwarfism (Denis et al., 2017). The *WOX* genes of the ancient clade in other species functioned similarly to that in Arabidopsis (Tvorogova et al., 2021). The intermediate clade contains four *AtWOX* genes (*AtWOX8*, *AtWOX9*, *AtWOX11*, and *AtWOX12*) (Deveaux et al., 2008; Tvorogova et al., 2021). The *AtWOX8* and *AtWOX9* co-regulate the development of the apical-basal polarity axis (Wu, Chory & Weigel, 2007; Lie, Kelsom & Wu, 2012). However, the orthologs of *AtWOX9* in other species are verified participating in the inflorescence development (Tvorogova et al., 2021). The *AtWOX11* and *AtWOX12* have similar functions in regulating the callus formation and development of adventitious roots (Liu et al., 2014a). The modern/WUS clade is the largest clade which includes eight *WOX* genes (*AtWUS* and *AtWOX1*-*7*) in Arabidopsis (Deveaux et al., 2008). Besides the homeodomain, *WOX* genes in the modern/WUS clade also have other conserved domains, such as the WUS motif or EAR domain (ERF-associated amphiphilic repression) (Deveaux et al., 2008; Wu, Li & Kramer, 2019). *WOX* genes in the modern/WUS clade participate in the regulating developments of various types of meristems (Tvorogova et al., 2021). In summary, the *WOX* family plays a central role in maintaining different types of meristems, regulating the formation of plant organs, and controlling cell proliferation and differentiation (Tvorogova et al., 2021).

Kiwifruit becomes a popular fruit worldwide owing to its high vitamin C content and abundant minerals (Cheng et al., 2004; Stonehouse et al., 2013). Kiwifruit belongs to the *Actinidia* genus, including 54 species and 75 taxa (Tang et al., 2019). The whole genome of the *A. chinensis* (Ac) and *A. eriantha* (Ae) has been reported (Pilkington et al., 2018; Tang et al., 2019). These two species are different in many vital traits, especially flowering time (Pilkington et al., 2018; Tang et al., 2019). The *WOX* gene family is verified to affect plant flowering and development (Tvorogova et al., 2021), whereas systematic investigations and functional analyses of the *WOX* gene family have not been reported in kiwifruit.

In the present study, we comprehensively identified the *WOX* gene family from *A. chinensis* and *A. eriantha* genome. We firstly reported the gene structure, motif compositions, chromosomal distributions of the *WOX* gene family for these two kiwifruit species. Further, we analyzed and compared the phylogenetic relationships and evolution patterns of the *WOX* gene family for the two kiwifruit species. *Cis*-elements analysis and expression patterns in different tissues and under different stress conditions were performed. Our results provided critical information on the structure characters, evolution patterns, and potential function of the *WOX* genes in the two kiwifruit species.

## Materials & methods

### Gene identification and analysis

The whole-genome sequences and protein sequences of the two kiwifruit species (*A. chinensis* and *A. eriantha*) were obtained from the Kiwifruit Genome Database (http://kiwifruitgenome.org/). The WOX protein sequences of *Arabidopsis* and rice were collected from the PlantTFDB v4.0 (http://planttfdb.gao-lab.org/). The local BLAST tool was used to construct the protein database of the two kiwifruit species. The combined protein sequences of *Arabidopsis* and rice WOX protein were used to query the kiwifruit protein database by the BLASTp. The candidate WOX proteins in kiwifruit were identified by BLASTp search scores of ≥ 100 and an e-value of ≤ 1 × e^−10^. The Conserved Domain Database (CDD) (https://www.ncbi.nlm.nih.gov/Structure/cdd/cdd.shtml) and the simple modular architecture research tool (SMART) (http://smart.embl.de/) were used to confirm the homeodomain of the candidate WOX protein, and the candidate WOX protein harboring the homeodomain were obtained and used for further analysis.

### Analysis of kiwifruit WOX protein structure

The protein length, theoretical isoelectric point (pI), grand average of hydropathicity (GRAVY), and molecular weight (MW) of the *WOX* gene family in the two kiwifruit species were computed using the ProtParam on ExPASy server (http://web.expasy.org/protparam/). The subcellular localization of kiwifruit WOX proteins was predicted using the online web software CELLO (v2.5, http://cello.life.nctu.edu.tw/).

### Gene structure, motif features, and cis-elements analysis

The genome sequences and coding sequences of the *WOX* genes of the two kiwifruit species were extracted. The gene structures of *WOX* genes were investigated using the Gene Structure Display Server (GSDS 2.0, http://gsds.cbi.pku.edu.cn/). The conserved motifs of WOX proteins were identified using MEME (http://meme-suite.org/tools/meme) with a maximum of 10 motifs (Bailey et al., 2009). To analyze *cis*-elements involved in regulating *WOX* genes, we extracted the 1,500-bp upstream promoter regions of each *WOX* gene, and predicted *cis*-elements with the PlantCARE database (http://bioinformatics.psb.ugent.be/webtools/plantcare/html/) (Lescot et al., 2002).

### Phylogenetic analysis of WOX proteins

The multiple sequence alignments of WOX proteins from *Arabidopsis thaliana*, *Oryza sativa*, *A. chinensis*, and *A. eriantha* were performed using ClustalX with default parameters (Larkin et al., 2007). The phylogenetic tree was constructed by MEGA X software using the neighbor-joining (NJ) method with 1,000 bootstrap replicates (Kumar et al., 2018).

### Gene duplication and synteny analysis

The genome location of *WOX* genes was extracted from the corresponding GFF file using an in-house Perl script, and the chromosomal distributions were rendered using MapGene2 Chrome (http://mg2c.iask.in/mg2c_v2.0/). The duplication patterns of kiwifruit *WOXs* were identified using the MCScanX software with default parameters (Wang et al., 2012). The synonymous (Ks) and nonsynonymous (Ka) mutation rates of the duplicated *WOX* gene pairs were calculated using TBtools software (Chen et al., 2020). The syntenic analysis of kiwifruit *WOXs* was conducted using the MCScanX software with default parameters to produce the collinearity blocks across the whole genome (Wang et al., 2012). The collinearity gene pairs of kiwifruit *WOXs* were visualized using TBtools (Chen et al., 2020).

### Expression analysis of kiwifruit WOXs

To investigate the expression patterns of kiwifruit *WOXs* in different tissues, developmental stages, or under stress treatments, we collected nine published RNA-seq data including those from leaves, roots, stems, different fruit developmental stages, fruits treated with or without ethylene, leaves infected with pathogens (PRJNA514180, PRJNA187369, PRJNA277383, PRJNA328414, PRJNA436459, PRJDB5543, and PRJNA535344) from NCBI (https://www.ncbi.nlm.nih.gov/). We further re-analyzed these transcriptome data using genomes of the *Actinidia chinensis* ‘Red5’ cultivar and *A. eriantha* white cultivar as reference genome (Pilkington et al., 2018; Tang et al., 2019). The reads alignment was performed using the HISAT2 v2.0.1 (Kim et al., 2019), and the transcripts were assembled and quantified using the STRINGTIE v2.1.5 (Pertea et al., 2015).

## Results

### Genome-wide identification and classification of kiwifruit WOXs

**Figure 1:**
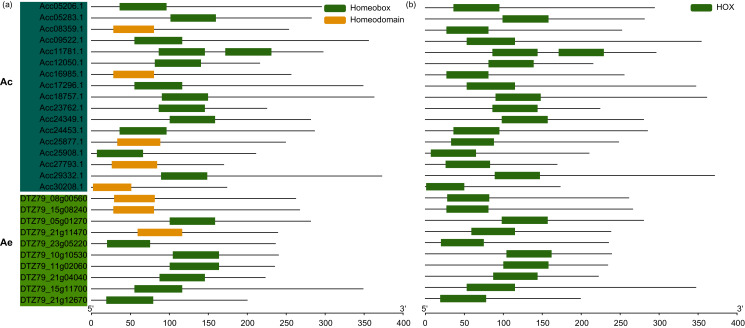
Conserved domain of *WOX*. Conserved domain of kiwifruit *WOX* gene predicted by Pfam (A) and SMART (B). Ac, *A. chinensis*; Ae, *A. eriantha*.

To identify potential *WOX* family members in kiwifruits, WOX protein sequences from Arabidopsis were used as queries in BLASTp homology search against Ac and Ae genomes. Totaly, we identified 17 and 10 putative WOXs from Ac and Ae genomes, respectively (Fig. 1 and Table S1). The homeodomain (PF00046 and SM00389) was verified by using Pfam and SMART databases (Fig. 1). The amino acid length of both *AcWOXs* and *AeWOXs* proteins varied greatly from 199 a.a (*AcWOX11a*) to 354 a.a (*AcWOX3a*) and from 169 a.a (*AeWOX4c*) to 371 a.a (*AeWOX10*) (Table 1; Table S1). The predicted molecular weight of AcWOX and AeWOX proteins ranged from 22.3 to 39.2 kDa and from 19.4 to 42.2 kDa (Table 1). Moreover, the theoretical isoelectric point (pI) ranged from 5.30 to 9.75 for AcWOXs and from 5.66 to 9.76 for AeWOXs (Table 1). The subcellular localization of kiwifruit WOX proteins was predicted, and all of AcWOX and AeWOX proteins were localized in the nuclear (Table 1).

**Table 1:** Characteristics of WOX proteins.

Species	Genes	Genome ID	Protein Length (aa)	MW (Da)	pI	GRAVY	Predicted Localiaztion
*A. chinensis*	AcWUS1a	Acc05206.1	239	27,182.38	9.08	−0.876	Nuclear
	AcWOX13a	Acc05283.1	266	29,050.87	5.49	−0.448	Nuclear
	AcWOX11a	Acc08359.1	199	22,315.54	9.75	−0.808	Nuclear
	AcWOX9a	Acc09522.1	280	32,003.07	5.87	−0.974	Nuclear
	AcWOX4a	Acc11781.1	222	25,554.82	9.51	−0.909	Nuclear
	AcWOX4b	Acc12050.1	261	28,424.23	5.39	−0.423	Nuclear
	AcWOX11b	Acc16985.1	238	27,037.52	5.30	−1.021	Nuclear
	AcWOX9b	Acc17296.1	235	26,266.99	9.47	−0.829	Nuclear
	AcWOX1a	Acc18757.1	347	37,969.27	8.30	−0.461	Nuclear
	AcWOX4c	Acc23762.1	236	27,155.85	5.70	−1.118	Nuclear
	AcWOX13b	Acc24349.1	294	32,910.35	5.79	−0.980	Nuclear
	AcWUS1b	Acc24453.1	281	32,134.20	5.79	−0.996	Nuclear
	AcWOX2	Acc25877.1	252	27,361.96	5.55	−0.466	Nuclear
	AcWOX3a	Acc25908.1	354	39,260.64	8.28	−0.606	Nuclear
	AcWOX5	Acc27793.1	296	34,123.19	9.46	−0.922	Nuclear
	AcWOX1b	Acc29332.1	215	24,485.44	9.05	−0.817	Nuclear
	AcWOX3b	Acc30208.1	255	27,862.43	5.38	−0.567	Nuclear
*A. eriantha*	AeWOX13	DTZ79_05g01270	361	41,243.21	6.74	−0.871	Nuclear
	AeWOX11	DTZ79_08g00560	224	25,913.02	9.20	−0.970	Nuclear
	AeWOX4a	DTZ79_10g10530	280	31,890.12	5.66	−0.907	Nuclear
	AeWOX4b	DTZ79_11g02060	285	31,718.21	6.32	−0.925	Nuclear
	AeWOX12	DTZ79_15g08240	248	27,951.01	9.76	−0.929	Nuclear
	AeWOX9	DTZ79_15g11700	210	24,383.34	8.58	−0.769	Nuclear
	AeWOX4c	DTZ79_21g04040	169	19,432.64	9.16	−0.785	Nuclear
	AeWOX10	DTZ79_21g11470	371	42,184.49	9.04	−0.875	Nuclear
	AeWUS1	DTZ79_21g12670	173	20,018.59	6.78	−0.611	Nuclear
	AeWOX2	DTZ79_23g05220	200	22,510.43	6.84	−0.615	Nuclear

**Note:**

Protein composition and physiochemical characteristics of kiwifruit WOX proteins.

### Phylogenetic and molecular evolution analysis of kiwifruit WOXs

**Figure 2:**
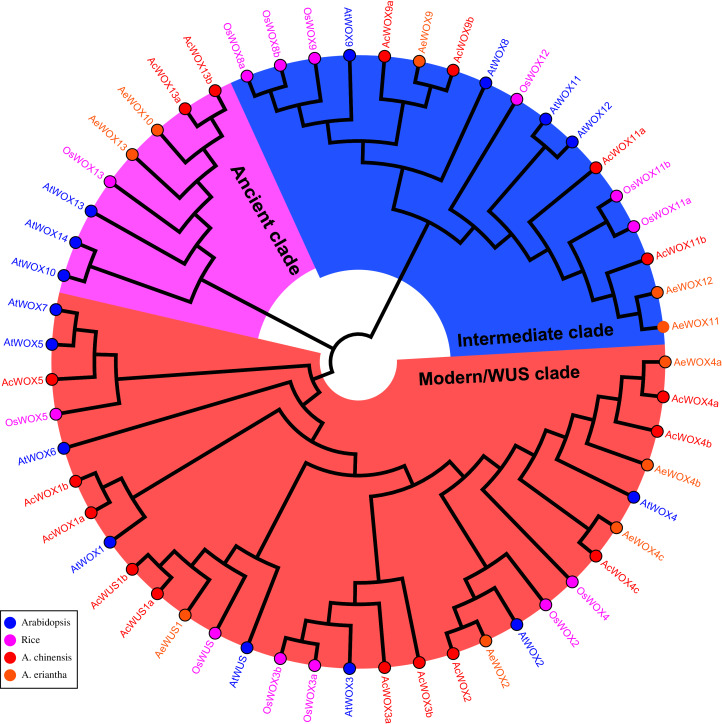
Phylogenetic tree of WOX. Phylogenetic tree of the WOX proteins. The full-length WOX protein sequences from rice (*Os*, pink gene name and circles), *Arabidopsis* (*At*, blue gene name and circles), *A. chinensis* (Ac, red gene name and circles) and *A. eriantha* (Ae, orange gene name and circles) were aligned using ClustalX 2.0 with default parameters. Then, the unrooted phylogenetic tree was constructed using MEGA X and the Neighbour-Joining method. The ancient clade, intermediate clade, and modern/WUS clade were highlighted using pink, blue, and light red sectors, respectively.

To explore the phylogenetic relationship and evolutionary pattern of *WOX* genes in kiwifruit, the neighbor-joining (NJ) tree was constructed using the full-length protein sequences of the identified 17 *AcWOXs*, 10 *AeWOXs*, and previously published 15 *AtWOXs* and 10 *OsWOXs* (*WOX* genes from Arabidopsis and rice). Consistent with previous reports in *Arabidopsis*, rice, and other species (Nardmann & Werr, 2012; Lin et al., 2013; Tang et al., 2017; Shafique Khan et al., 2021), both *AcWOXs* and *AeWOXs* were classified into three clades (the ancient clade, the intermediate clade, and the modern/WUS clade) (Fig. 2). Eleven of 17 *AcWOXs* and five of 10 *AeWOXs* were assigned in the modern/WUS clade, and the ancient clade contained the least number of both *AcWOX* and *AeWOX* genes (two *AcWOXs* and two *AeWOXs*) (Fig. 2). The intermediate clade had four *AcWOXs* and three *AeWOXs* (Fig. 2). *AcWOXs* and *AeWOXs* grouped with different *WOX* genes in Arabidopsis and rice indicated that both *AcWOXs* and *AeWOXs* probably had abundant diversified functions similar to *WOX* genes in Arabidopsis and rice (Fig. 2).

### Chromosomal localization and structure analysis of kiwifruit WOXs

**Figure 3:**
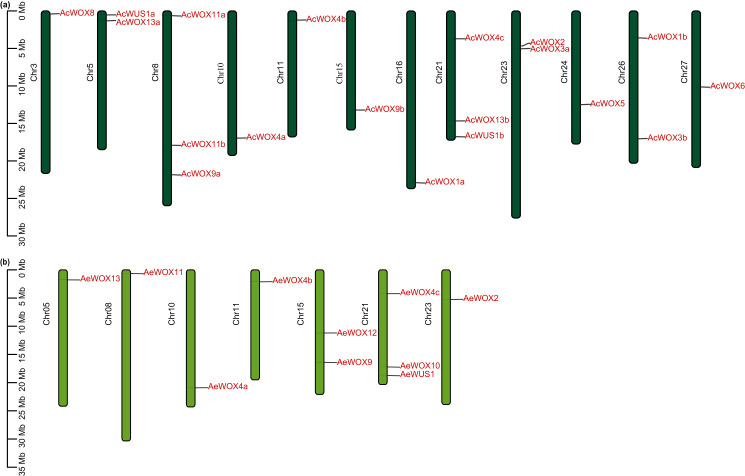
Chromosome location of WOX. Distribution of *WOX* genes in Ac (A) and Ae (B) genomes.

The 17 *AcWOX* genes were randomly distributed on 12 chromosomes of Ac (Fig. 3A), of which chromosome 8 and 21 included the most abundant *WOX* genes (three *AcWOX* genes), followed by three chromosomes (chr 5, 23, and 26) containing two genes and the other seven chromosomes (chr 3, 10, 11, 15, 16, 24, and 27) containing one *AcWOX* gene (Fig. 3A and Table S1). Similarly, the 10 *AeWOX* genes were unevenly distributed on seven chromosomes. Chr 21 and 15 contained three and two *AeWOX* genes, separately, and each of the other five chromosomes (chr 5, 8, 10, 11, and 23) possessed one *AeWOX* gene (Fig. 3B and Table S1).

**Figure 4:**
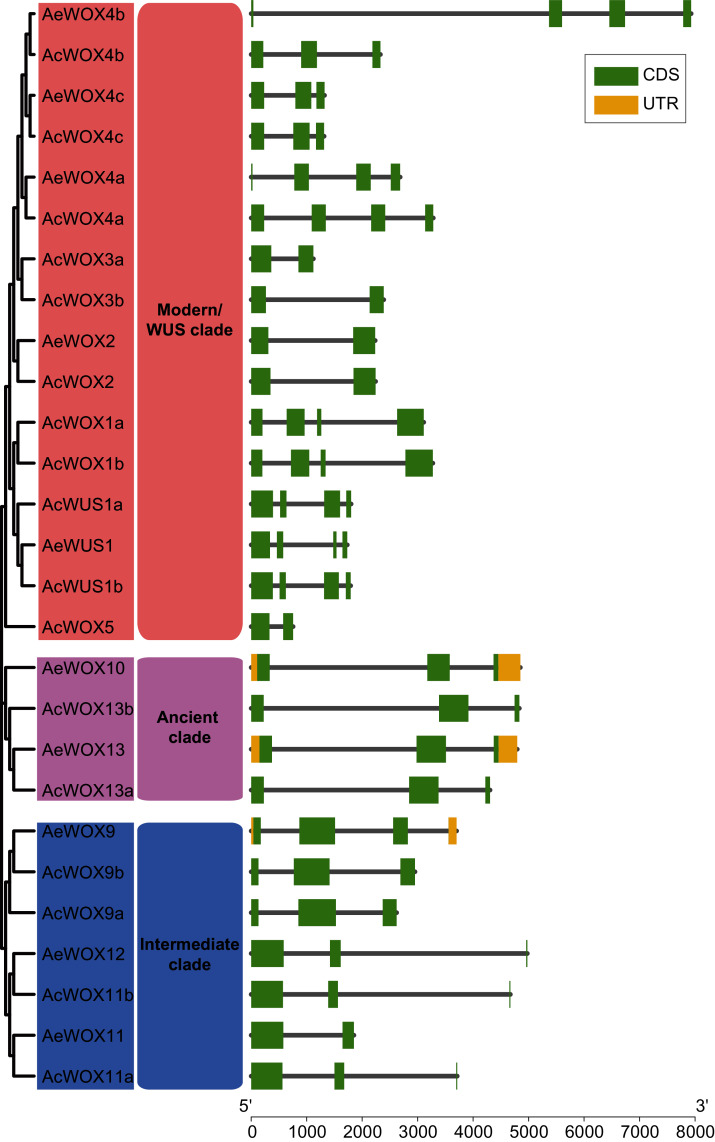
Structures of WOX genes. Exon-intron structures of *WOX* genes in two kiwifruit species. The left panel indicated the phylogenetic tree containing AcWOX and AeWOX proteins; the middle panel showed three clades; the right panel showed exon-intron structures of kiwifruit *WOX* genes. The green rectangle, the yellow rectangle, and the regular line represent exons, UTRs, and introns, respectively.

The exon-intron structure is a primary evolutionary feature of a gene family and provides a clue for function diversification and classification (Shiu & Bleecker, 2003). The exon number of *AcWOXs* and *AeWOXs* varied from two to four (Fig. 4). In terms of intron number and exon length, both *AcWOXs* and *AeWOXs* in the ancient clade were conserved for exon-intron structures (Fig. 4). In the intermediate clade, exon numbers of *AeWOXs* varied from two to four. In contrast, the *AcWOXs* in this clade had a fixed exon number, indicating that *WOX* genes had undergone varied evolutionary patterns in the two kiwifruit species. In each species, *WOX* genes in the same clade were more similar than those among different clades. Moreover, the exon-intron structure of both *AcWOX* and *AeWOX* genes diverged more in the modern/WUS clade, and this may result in the functional diversification of *WOXs* in this clade in two kiwifruits (Fig. 4).

### Domain architecture and conserved motifs analysis of kiwifruit WOXs

**Figure 5:**
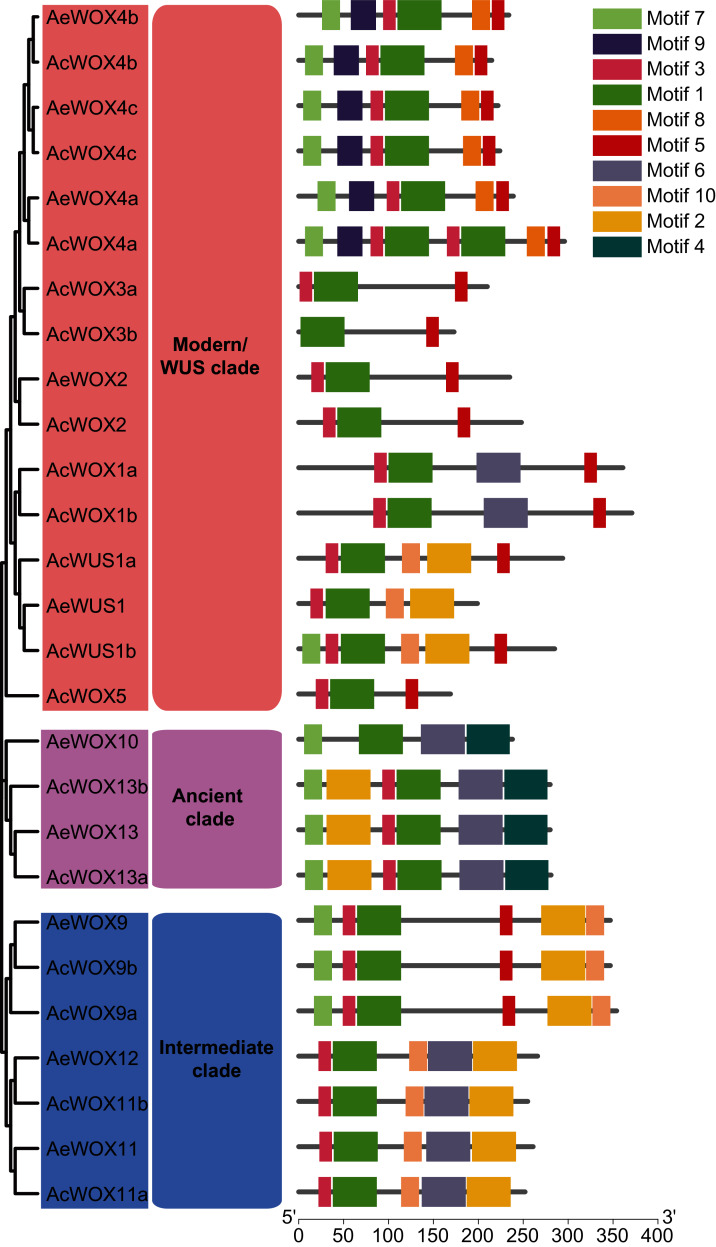
Motifs of WOX. Motif architectures of kiwifruit WOX proteins. The left panel indicated the phylogenetic tree of AcWOX and AeWOX protein sequences; the middle panel showed the defined clades; the right panel showed motif architectures of WOX proteins. Rectangles with different colors represented different motifs.

To infer the conserved motif architectures of kiwifruit *WOXs*, the motif compositions of entire sequences were predicted by MEME (Bailey et al., 2009) (Fig. 5 and Fig. S1). In total, 10 conserved motifs were identified for the kiwifruit *WOXs* and designated as motif 1–10 (Fig. S1). The motif number of the *WOX* gene ranged from two to eight in each kiwifruit (Fig. 5). Within the modern/WUS clade, the motif number varied greatly from two to eight, while motif numbers in the intermediate clades (five or six) and ancient clades (four or six) were stable (Fig. 5). All *WOX* genes in the two kiwifruit species contained motif 1, and we confirmed that motif 1 was the homeodomain with Pfam and SMART databases (Fig. 5). Furthermore, we found that motif 1 sequences in kiwifruit *WOX* genes were highly conserved (Fig. S2). Two and one clade-specific motifs were found in modern/WUS clade *WOXs* (motif 8 and 9) and ancient clade *WOXs* (motif 4), respectively (Fig. 5). Similar to results of exon-intron structure, closely phylogenetically related *WOX* genes showed conserved motif structures, including motif number and organization, which indicated similar functions among them (Fig. 5).

### Synteny and duplicated gene analysis of kiwifruit WOXs

**Figure 6:**
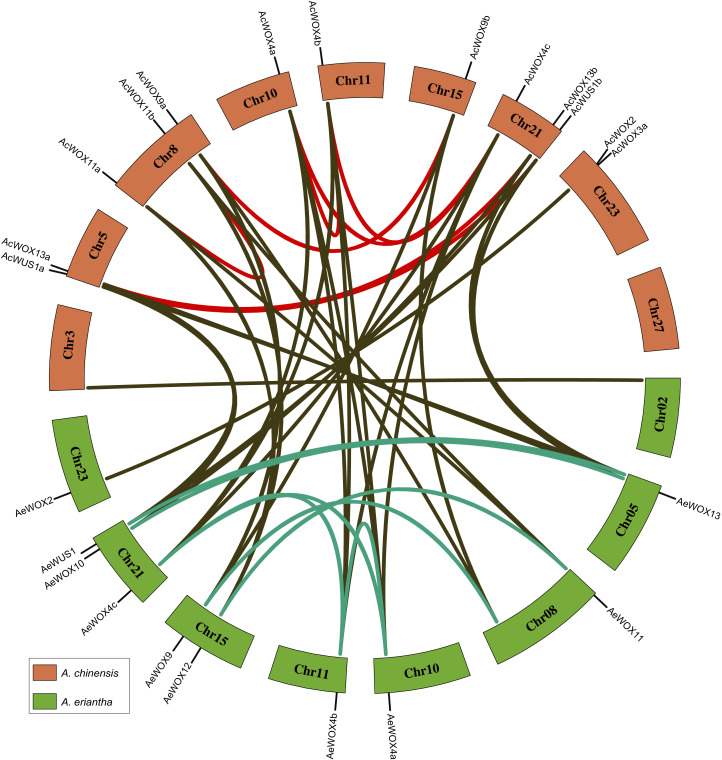
Synteny relationship of WOX. Chromosome distribution and synteny relationship of *WOX* genes in two kiwifruit species. The light red and green bars indicated chromosomes for Ac and Ae, respectively. The syntenic gene pairs were connected by lines with different colors.

Gene duplication and loss were the main evolutionary forces driving the expansion or contraction of gene families, and duplicated genes could result in gene redundancy or new functionalization (Tang et al., 2020). To visualize the synteny relationships among homologous *WOX* genes and infer gene duplication events, we conducted a collinearity analysis by using MCScanX (Wang et al., 2012). Accordingly, we determined that gene pairs belonging to five types of gene duplication (singleton duplication (SD), dispersed duplication (DD), proximal duplication (PD), tandem duplication (TD), and whole-genome duplication (WGD)). We identified eight and five pairs of genes that resulted from duplication in Ac and Ae kiwifruit, respectively (Fig. 6 and Table 2). All duplicated gene pairs belonged to the modern/WUS and the intermediate clades, indicating these kiwifruit *WOXs* diverged considerably and had more potential to be new functionalized than that in the ancient clade (Fig. 6 and Table 2). All duplicated gene pairs were produced by the whole-genome duplication, indicating that the WGD accounted for the expansion of kiwifruit *WOX* families (Table 2).

**Table 2:** WOX duplication events.

Species	Duplicate pairs	Ka	Ks	Ka/Ks	Duplication
*A. chinensis*	AcWOX4a/AcWOX4b	0.06322	0.23011	0.27473	WGD
	AcWOX4a/AcWOX4c	0.18728	0.71925	0.26038	WGD
	AcWOX4b/AcWOX4c	0.16292	0.70114	0.23237	WGD
	AcWOX9b/AcWOX9a	0.06494	0.20962	0.30979	WGD
	AcWUS1b/AcWUS1a	0.02622	0.15924	0.16464	WGD
	AcWOX13b/AcWOX13a	0.03055	0.22185	0.13769	WGD
	AcWOX3a/AcWOX3b	0.15948	0.52930	0.30131	WGD
	AcWOX11a/AcWOX11b	0.04509	0.15773	0.28589	WGD
*A. eriantha*	AeWOX13/AeWOX10	0.02696	0.22560	0.11951	WGD
	AeWOX11/AeWOX12	0.05723	0.16531	0.34620	WGD
	AeWOX4a/AeWOX4b	0.12001	0.35079	0.34212	WGD
	AeWOX4a/AeWOX4c	0.18328	0.68682	0.26686	WGD
	AeWOX4b/AeWOX4c	0.20150	0.78790	0.25574	WGD

**Note:**

WOX duplication events identified in kiwifruits.

To estimate the selection pressure that kiwifruit *WOXs* experienced after the gene duplication, we calculated the Ka/Ks ratios, ratios of the rate of nonsynonymous substitution (Ka) to the rate of synonymous substitution (Ks) (Table 2 and Table S2). Generally, the Ka/Ks value reflects the selection pressure during evolution (Ka/Ks = 1: neutral selection; Ka/Ks > 1: positive selection; Ka/Ks < 1: purifying selection) (Alvarez et al., 2018). In this study, Ka/Ks ratios of *WOXs* ranged from 0.096 to 0.436, with an average of 0.259 (Table 2 and Table S2). These results suggested that purifying selection was the primary evolutionary force acting on kiwifruit *WOXs*.

### Cis-element analysis of promoter regions of kiwifruit WOXs

**Figure 7:**
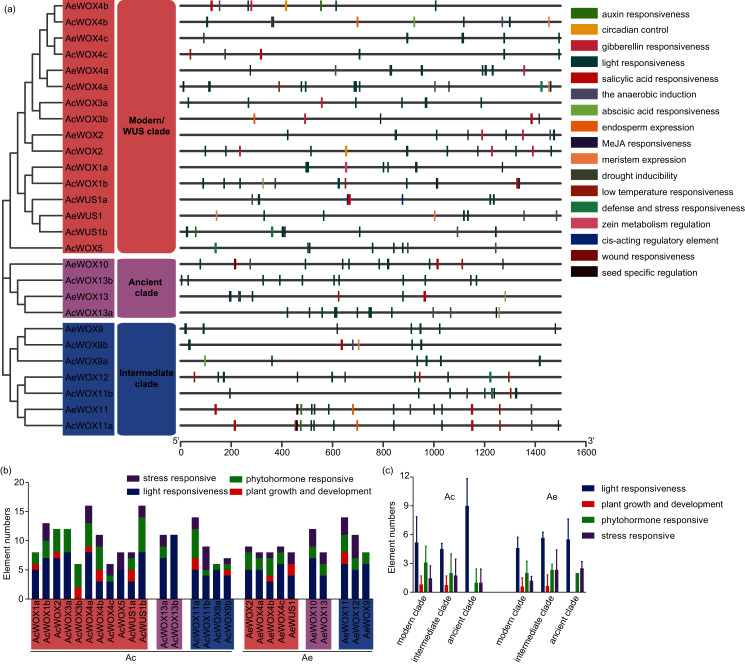
*Cis*-elements of WOX. *Cis*-elements analysis in the promoter regions of kiwifruit *WOX* genes. (A) The *cis*-element architectures in the 1,500-bp promoter regions of kiwifruit *WOXs*. Rectangle with different colors represented different *cis*-elements. (B) The number of *cis*-elements in the promoter region of each kiwifruit *WOX* gene. (C) The average number of *cis*-elements for each clade was showed.

The *cis*-element plays a crucial control in transcriptional regulation and significantly affects gene function (Nakashima, Ito & Yamaguchi-Shinozaki, 2009). The 1,500-bp upstream region of each kiwifruit *WOX* gene was extracted and employed for the *cis*-element prediction. Totally, 17 functional *cis*-elements were retained, and the core promoter elements, such as TATA-box and CAAT-box, were presented in all promoters of kiwifruit *WOXs*. The 17 functional *cis*-elements were classified into four subfamilies, including light responsiveness, plant growth and development, phytohormone responsive, and stress-responsive subfamily (Fig. 7A). Elements of the light responsiveness subfamily were the most abundant presented in the promoter regions of kiwifruit *WOX* genes of the two species, indicating that light could significantly affect expression patterns of kiwifruit *WOXs* and gene function (Fig. 7B). The number of light responsiveness *cis*-element in all kiwifruit *WOXs* ranged from 0 (*AcWOX3b*) to 11 (*AcWOX13b*) (Figs. 7A and 7B). The number of the light responsiveness *cis*-element within *WOXs* differed between two kiwifruit species (Fig. 7C). The *cis*-element organizations for the duplicated gene pairs were evolved divergently (Fig. 7A), suggesting the specific expression patterns and new functionalization for the duplicated gene pairs. However, the *cis*-element arrangements of the orthologous *WOX* gene pairs for the two species were highly similar, indicating that the orthologous *WOX* gene pairs possibly had similar functions (Fig. 7A).

### Expression patterns of kiwifruit WOXs

**Figure 8:**
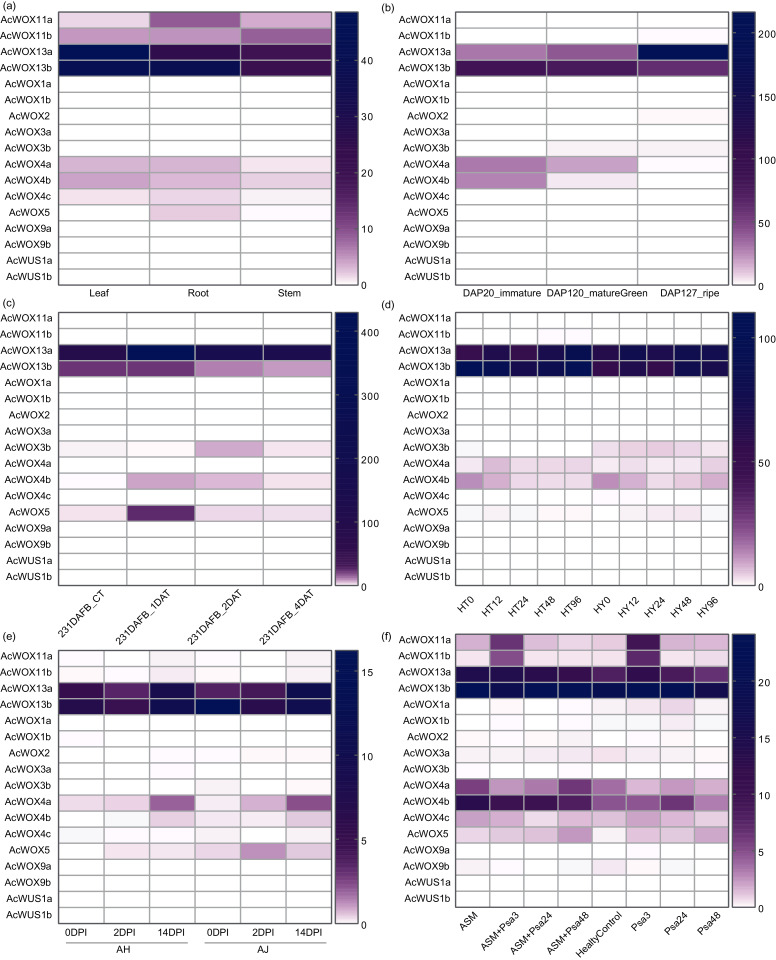
Expression patterns of WOX. Expression profiles of *AcWOX* genes in different tissues, fruit developmental stages, and under different treatments. The heatmap indicated the FPKM (fragments per kilobase of exon model per million mapped reads) values of *AcWOX* genes. (A) Expression profiles of *AcWOXs* in three tissues. (B) Expression profiles of *AcWOXs* in three fruit developmental stages. DAP, days after pollination. (C) Expression profiles of *AcWOXs* in samples treated with ethylene. DAFB, days after full bloom of fruit; DAT, day after treated with ethylene. (D) Expression profiles of *AcWOXs* in two kiwifruit cultivars infected with Psa. HT and HY represented resistant and susceptible cultivar, respectively. The number following the cultivar name showed hours post the Psa invasion. (E) Expression profiles of *AcWOXs* in two susceptible cultivars to the invasion of Psa. DPI, days post-infection. (F) Expression profiles of *AcWOXs* in samples with or without Acibenzolar-S-methyl (ASM) treatment during the Psa infection. Healtycontrol represented samples without any treatment or infection. The number presented in the sample names indicated hours post the Psa infection.

Firstly, we investigated the expression patterns of *AcWOX* genes in three tissues (leaf, root, and stem) based on transcriptome data (Fig. 8A). Our results showed that *AcWOX* genes had highly tissue-specific expression patterns (Fig. 8A). *AcWOX13a* and *AcWOX13b* highly expressed in all three tissues but more abundant in leaf and root, indicating that expression of *AcWOX13a/13b* might be essential for the leaf and root (Fig. S3). The other four *AcWOXs*, including *AcWOX4a/4b/11a/11b*, were expressed lowly in three tissues (Fig. S3). While the *AcWOX5* is expressed explicitly in the root (Fig. 8A and Fig. S3). We further analyzed the expression profiles of *AcWOX* genes during fruit development. Four *AcWOX* genes highly expressed in fruits, and they gradually increased (*AcWOX13a*) or decreased (*AcWOX4a/4b/13b*) their expressions along with fruit development (Fig. 8B). These indicated that these four *AcWOXs* might affect fruit development differently (Fig. S3B).

To further confirm whether the expression of *WOX* genes was influenced by hormonal treatments and biotic stresses, we analyzed transcriptomic data from Ac fruits which treated with ethylene (Gunaseelan et al., 2019) or invaded with *Pseudomonas syringae* pv. *actinidiae* (Psa) which was the pathogen of kiwifruit bacterial canker disease (Michelotti et al., 2018). Totally, five *AcWOX* genes significantly responded to ethylene treatments (Fig. 8C). Among them, four genes (*AcWOX3b/4b/5/13a*) were upregulated by ethylene treatment and gradually reduced their expressions after the treatment, and the *AcWOX13a* had the highest expression level (Fig. S3C). Besides, *AcWOX13b* was downregulated by the ethylene treatment (Fig. S3C).

We detected *AcWOX* gene expression profiles in response to Psa infection with three sets of transcriptomic comparisons, *i.e*., (a) comparative transcriptomes of the resistance cultivar and the susceptible cultivar (Fig. 8D); (b) two susceptible cultivars (Fig. 8E); (c) with or without Acibenzolar-S-methyl (ASM) treatment (Fig. 8F). In the first comparison, we identified five highly expressed *AcWOX* genes with varied transcriptional levels among different infection times (Fig. 8D). *AcWOX13a* and *AcWOX13b* displayed abundant expression in both cultivars and showed higher expressions in the resistant variety (HT) than in the susceptible variety (HY) (Fig. 8D and Fig. S3D), indicating that *AcWOX13a* and *AcWOX13b* might affect kiwifruit resistance to Psa. Similar to the results in the first set, transcriptional levels of *AcWOX13a* and *AcWOX13b* were higher than others under the infection of Psa in the second set of comparison (Fig. 8E and Fig. S3E). The third set of the comparison showed that ASM inoculated kiwifruits increased their expression of *AcWOX13a/13b/4a/4b* compared with samples without the ASM treatment (Fig. 8F and Fig. S3F).

## Discussion

The *WOX* gene family is the core regulator for forming the shoot apical meristem and embryonic development, stem cell maintenance, and various other developmental processes in plants (Ariel et al., 2007; Wu, Chory & Weigel, 2007; Deveaux et al., 2008; Shimizu et al., 2009; van der Graaff, Laux & Rensing, 2009; Palovaara et al., 2010). Genome-wide identification of the *WOX* gene family had been accomplished in several plants (Deveaux et al., 2008; Liu et al., 2014b; Cheng et al., 2014; Tang et al., 2017; Rahman et al., 2017; Li et al., 2018, 2020; Ramkumar et al., 2018; Shafique Khan et al., 2021). However, genome-wide characterization of the *WOX* gene family had not been conducted in kiwifruits. In the present study, we *in silico* identified the genome-wide *WOX* gene family in two kiwifruit species (Ac and Ae). Further, we compared the characters and evolutionary patterns of kiwifruit *WOXs* of the two species. In addition, we analyzed and compared *cis*-element organizations for promoter regions of kiwifruit *WOXs* identified in the two species. We also investigated the expression profiles of *AcWOXs* in different tissues and different fruit developmental stages and the influence of hormonal treatment and biotic stresses on *AcWOXs* expression.

Seventeen and 10 *WOX* genes were identified in Ac and Ae, respectively (Fig. 1 and Table 1). Compared to the *WOX* family in *Arabidopsis*, the Ac has more while Ae has fewer family members. Interestingly, no *WOX* genes from Ac and Ae are classified together with *AtWOX6*, and no *WOX* genes from Ae are classified together with *AtWOX1/5/7*. This may be due to the gene loss in the evolution of *Actinidia* species. The lost of *WOX6* homologous gene has also been reported in many plants including Orchidaceae species (Kanchan & Sembi, 2020; Victorathisayam et al., 2020), Solanaceae species (Li et al., 2018), and cucumber (Han et al., 2021). *AtWOX6* regulates ovule development and plays a role in freezing tolerance in Arabidopsis (Park et al., 2005). However, *WUS* and *WOX6* genes possess some similar functions and could be replaced mutually sometimes (Dolzblasz et al., 2016). *WOX1* and *WOX3* has been demonstated redundantly control leaves development in Petunia, Arabidopsis and tomatos (Vandenbussche et al., 2009; Nakata et al., 2018; Wang et al., 2021). *WOX6* and *WOX7* comprise close relatives of *WOX1* and *WOX5*, respectively, and are possibly due to the recent whole-genome duplication (Blanc & Wolfe, 2004). These suggest that *WOX* gene family members could be replaced, and it may make their losing in evolution possible. *WOX1* has been domenstrated that was possibly lost in monocots, where grass genomes manifested a *WOX3* duplication (Nardmann & Werr, 2013).

Duplicated *WOX* gene pairs in both Ac and Ae were entirely owing to WGD (Fig. 6 and Table 2). Genomic analyses verified that both Ac and Ae genomes experienced three ancient WGD events (Pilkington et al., 2018; Tang et al., 2019), which supported our results. However, the difference of *WOX* gene number between Ac and Ae indicated that the *WOX* gene family in Ac and Ae had undergone inconsistent evolutionary patterns (Pilkington et al., 2018; Tang et al., 2019). Together with the asymmetric gene losing in these two kiwifruit species, *WOX* gene family members are species-specific in Actinidia genus. In addition, we inferred that translocation, gene retention, and gene loss post whole-genome duplications were the evolutionary forces causing variations of the *WOX* gene number and distribution in Ac and Ae genomes. Signals of purifying selection were found for all *WOX* genes in both species, which indicated the crucial roles of kiwifruit *WOXs*.

Homeodomain, a domain that contains a helix-loop-helix-turn-helix structure (Katoh & Standley, 2013), is conserved in the *WOX* gene family among different species and thus maintains the functional integrity of *WOX* genes. Except for the homeodomain, we additionally identified nine conserved motifs in kiwifruit *WOXs* (Fig. 5). Clade-specific or subclade-specific motifs in kiwifruit *WOXs* genes suggested the functional differentiation among *WOXs* in different clades (Fig. 5). However, gene structures and conserved motif organizations of most kiwifruit *WOXs* belonging to the same subclade were consistent. The *cis*-element analysis of promoter regions of kiwifruit *WOXs* indicated that the *cis*-element organizations of kiwifruit *WOXs* were extremely varied, even for those in the same subclade (Fig. 7). We inferred that the *cis*-element organizations of *WOXs* belonging to the same subclade regulated the functional divergence of those *WOXs* by regulating their expression patterns, which was verified by the results of expression analysis (Fig. 8). In total, gene structure, motif organization, and *cis*-element arrangement precisely controlled the functional divergent of kiwifruit *WOXs*.

Promoters are regions of DNA that initiate transcription of particular genes. They play prominent roles in the temporal and spatial regulation of gene expression. In the present study, a series of development-, hormone-, response- and stress- related *cis*-elements were detected in the promoter of kiwifruit *WOXs* (Fig. 7). Each *WOX* gene harbours at least six *cis*-elements belonging to at least two different functional subfamilies, except *AcWOX13b* which has 11 light responsiveness *cis*-elements (Fig. 7). Accumulating evidence has demonstrated that plant stress resistance is regulated by phytohormones (Chaiprasongsuk et al., 2018; Ditengou et al., 2018). We suspect that expression of *WOX* genes were influenced by stress, and it was uncovered by RNA-seq results in Ac. However, the relationship between the stress resistance and phytohormones needs more evidences.

Gene expression patterns in different tissues or under different treatments can be used to identify the functions of genes. Expression patterns of *WOX* genes are different either between *A. thaliana* and *Populus tomentosa* (Liu et al., 2014b) or between *A. thaliana* and rice (Zhang et al., 2010). In this study, expression patterns of *WOX* genes in kiwifruits were different from those plants informed above (Fig. 8). These implied that the functions of *WOX* genes might be different among monocots and dicotyledones of herbal plants, woody plants and liane.

In *AcWOX* and *AeWOX* members, few genes were mainly expressed in specific organs, indicating that they may be involved in different development processes. In Ac kiwifruit, *AcWOX13a* and *AcWOX13b* expressed higher than other *WOXs*, and they often respond to stresses dramatically (Fig. 8). This indicated the pivotal role of *WOXs* of the ancient clade in kiwifruit development and stress resistance. Similar functioned *WOX* gene (*OsWOX11* and *OsWOX13*) has been reported in rice (Tvorogova et al., 2021). *OsWOX11*, a member of the intermediate clade, was the most reported, responding to abiotic stresses such as drought and cold reviewed in (Tvorogova et al., 2021). Minh-Thu reported that *OsWOX13*, an ancient clade *WOX* gene, enhances drought tolerance and triggers early flowering in rice (Shafique Khan et al., 2021). In poplar, over-expression of *PtoWOX13* led to an increased adventitious root (AR) number, deceased AR length or increased AR roughtnees (Liu et al., 2014b). In walnut, *JrWOX13* plays an important role in later root development (Chang et al., 2020). It is unclear whether *AcWOX13a* and *AcWOX13b* regulate the stress resistance *via* root development. Further study of the precise function of *AcWOX13a* and *AcWOX13b* will be needed in the future.

In summary, the present study firstly detected *WOX* genes from kiwifruit genomes. Furthermore, we identified the potential *WOXs* in response to hormonal treatments and biotic stresses. Our research will provide a foundation for accelerating the genetic breeding of kiwifruits.

## Conclusions

In conclusion, we performed genome-wide identification and characterization of the *WOX* gene family in *Actinidia chinensis* (Ac) and *A. eriantha* (Ae) and conducted a detailed investigation of their evolutionary relationship, genome organization, duplication events and expression profiles. In total, 17 and 10 *WOX* genes have been identified from Ac and Ae genomes. They were classified into three clades (the ancient clade, the intermediate clade, and the morden/WUS clade) based on the sequence alignment and phylogenetic analysis, which was corresponding to previous report of Arabidopsis. Gene structures and motif patterns showed that *WOX* members in the same clade displayed more similarly. Gene duplications and selective pressure analysis indicated that *WOX* genes in two kiwifruit species have undergone different evolution patterns after the shared genome duplication events. Subcellular localization revealed that all *WOX* genes were located inside the nucleus. Furthermore, expression patterns detected tissue-specific expressed genes and hormonal or abiotic-stress responding genes. Overall, *WOX* genes identified in Ac and Ae genomes provided insight into general characters, evolutionary patterns, and functional diversity of kiwifruit *WOXs*.

## Supplemental Information

10.7717/peerj.12348/supp-1 Supplemental Information 1 Logos of motifs. Sequence logos for the ten conserved motifs identified in the kiwifruit *WOX* gene family.

10.7717/peerj.12348/supp-2 Supplemental Information 2 Homeodomain. Sequence alignment of the homeodomain in the kiwifruit WOX proteins.

10.7717/peerj.12348/supp-3 Supplemental Information 3 Expression profiles of *WOX* genes. Expression profiles of *WOX* genes. *AcWOXs* with high expression levels in three tissues and three fruit developmental stages were shown in (a) and (b). DAP, days after pollination. *AcWOXs* with high expression levels in samples treated with ethylene was shown in (c). DAFB, days after full bloom of fruit; DAT, day after treated with ethylene. (d) showed *AcWOXs* with high expression levels in two kiwifruit cultivars infected with Psa. HT and HY represented resistance and susceptible cultivar, respectively. The number in cultivar names showed hours post the Psa invasion. (e) shows expression profiles of *AcWOXs* with high expression levels in two susceptible cultivars to the invasion of Psa. DPI, days post-infection. (f) was expression profiles of *AcWOXs* with high expression levels in samples with or without Acibenzolar-S-methyl (ASM) treatments during the Psa infection. Healtycontrol represents samples without ASM treatment and Psa infection. The number presented in sample names indicated hours post the Psa infection.

10.7717/peerj.12348/supp-4 Supplemental Information 4 Characteristics of kiwifruit WOX genes.

10.7717/peerj.12348/supp-5 Supplemental Information 5 Ka/Ks analysis for kiwifruit WOX gene pairs.

## Additional Information and Declarations

### Data Availability

The following information was supplied regarding data availability: Feng, Chen (2021): RNA-Seq analysis. figshare. https://doi.org/10.6084/m9.figshare.15134973.v1.
