# Supplementary figures and images for: In silico identification, characterization expression profile of WUSCHEL-Related Homeobox (WOX) gene family in two species of kiwifruit
Source: PeerJ. 2021 Oct 28;9:e12348. doi: 10.7717/peerj.12348 (PMC8557698; doi:10.7717/peerj.12348)

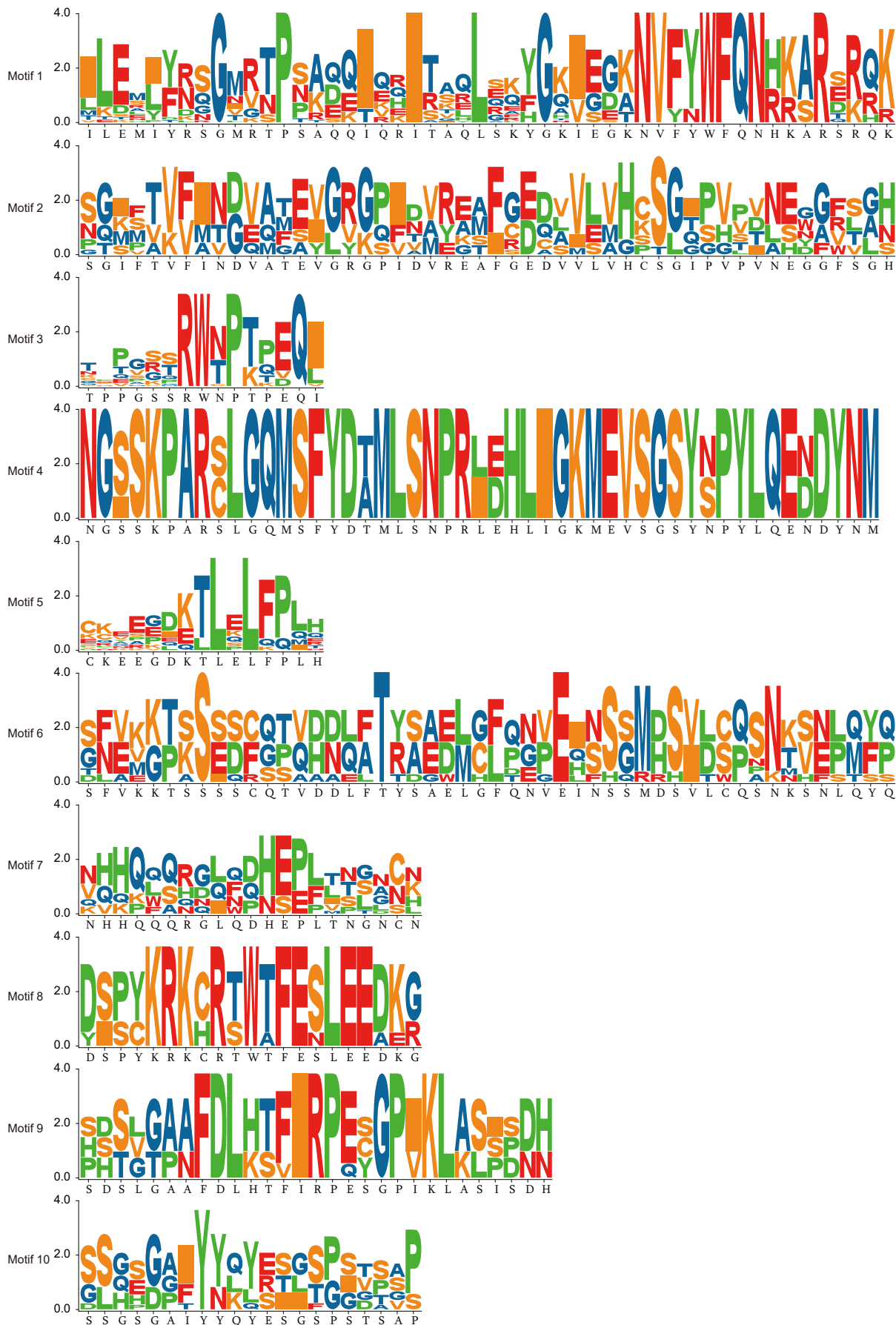

Supplement: Supplemental Information 1 — Sequence logos for the ten conserved motifs identified in the kiwifruit WOX gene family. [file peerj-09-12348-s001.pdf]

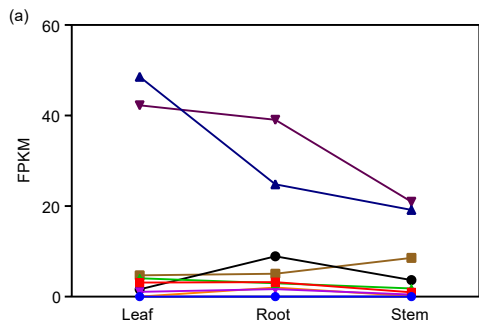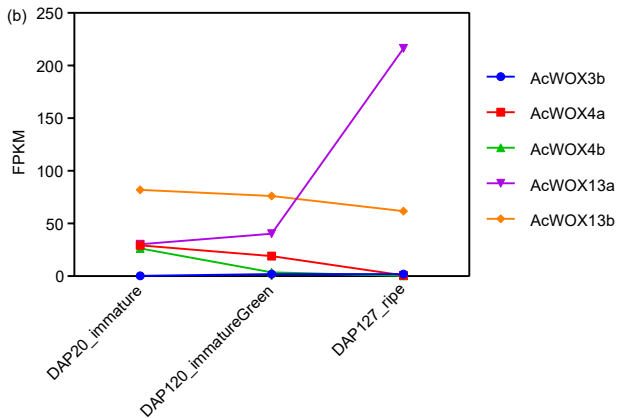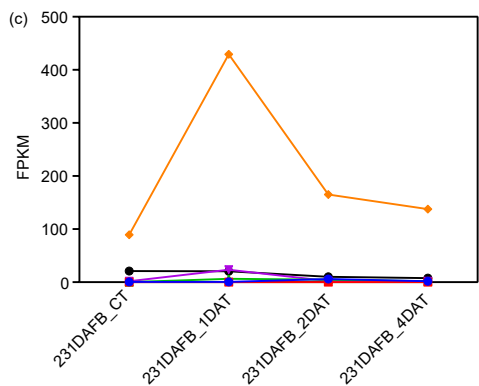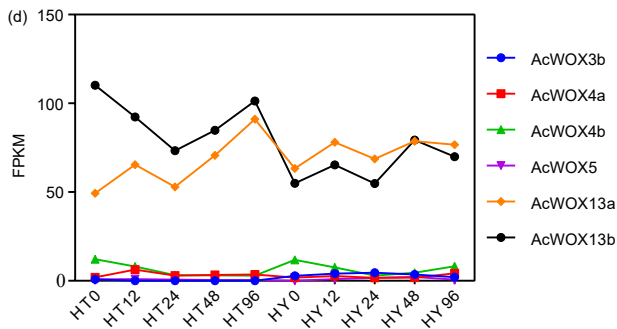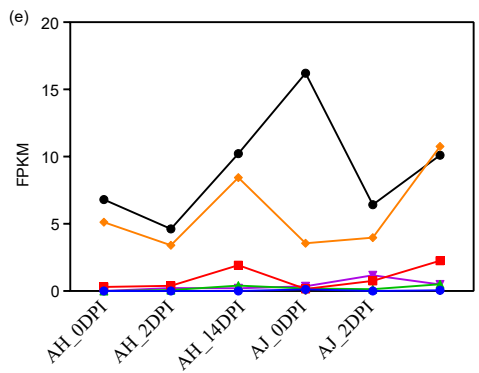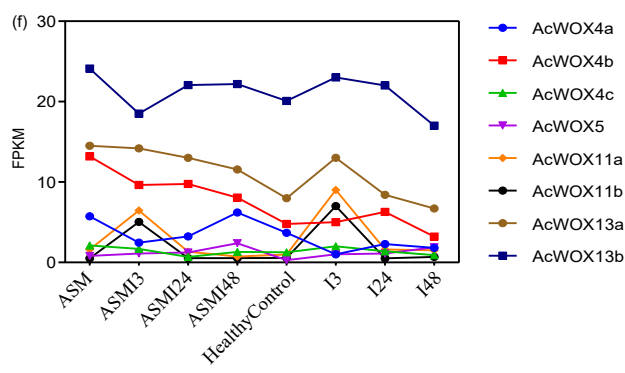

Supplement: Supplemental Information 3 — Expression profiles of WOX genes. AcWOXs with high expression levels in three tissues and three fruit developmental stages were shown in (a) and (b). DAP, days after pollination. AcWOXs with high expression levels in samples treated with ethylene was shown in (c). DAFB, days after full bloom of fruit; DAT, day after treated with ethylene. (d) showed AcWOXs with high expression levels in two kiwifruit cultivars infected with Psa. HT and HY represented resistance and susceptible cultivar, respectively. The number in cultivar names showed hours post the Psa invasion. (e) shows expression profiles of AcWOXs with high expression levels in two susceptible cultivars to the invasion of Psa. DPI, days post-infection. (f) was expression profiles of AcWOXs with high expression levels in samples with or without Acibenzolar-S-methyl (ASM) treatments during the Psa infection. Healtycontrol represents samples without ASM treatment and Psa infection. The number presented in sample names indicated hours post the Psa infection. [file peerj-09-12348-s003.pdf]
