# Supplementary material for: In silico identification, characterization expression profile of WUSCHEL-Related Homeobox (WOX) gene family in two species of kiwifruit
Source: PeerJ. 2021 Oct 28;9:e12348. doi: 10.7717/peerj.12348 (PMC8557698; doi:10.7717/peerj.12348)
Supplement: Supplemental Information 2 — Sequence alignment of the homeodomain in the kiwifruit WOX proteins. [file peerj-09-12348-s002.pdf]

```
AeWOX4b   ILEMLY-RSGMRTPNAQQIEHITAQLGKYGKIEGKNVFYWFQNHKARDRQK
AcWOX4b   ILEMLY-RSGMRTPNAQQIEHITVQLGKYGKIEGKNVFYWFQNHKARDRQK
AcWOX4a   ILEMLY-RSGMRTPNAQQIEHITTQLGKYGKIEGKNVFYWFQNHKARDRQK
AeWOX4a   ILEMLY-RSGMRTPNAQQIEHITTQLGKYGKIEGKNVFYWFQNHKARDRQK
AeWOX4c   ILEMLY-RGGMRTPNAQQIEQIAVQLEKYGKIEGKNVFYWFQNHKARERQK
AcWOX4c   ILEMLY-RGGMRTPNAQQIEQITVQLEKYGNIEGKNVFYWFQNHKARERQK
AcWOX1a   TLEELYR-QGTRTPSAEQIQHITAQLRRYGKIEGKNVFYWFQNHKARERQK
AcWOX3a   ILEEIY-RGGTRTPNASQIQQITAHLAFYGKIEGKNVFYWFQNHKARDRQK
AcWOX1b   TLKELYR-RGTRTPSADQIQHITSHLRRYGKIEGKNVFYWFQNHKARERQK
AcWUS1b   -LKDLYYNNGVRSPSAEQIQRISARLRQYGKIEGKNVFYWFQNHKARERQK
AcWUS1a   -LKDLYYNNGVRSPSAEQIQRISARLRQYGKIEGKNVFYWFQNHKARERQK
AeWUS1    -LKDLYYNNGVRSPSAEQIQRISARLRQYGKIEGKNVFYWFQNHKARERQK
AcWOX3b   -LEEMYTRGGIRTPNASQIQQITAYLSLYGKIEGKNVFYWFQNHKARDRQK
AcWOX9a   ILEAIFNS-GMVNPPRDEIRKIRAQLQEYGQVGDANVFYWFQNRKSRTKHK
AcWOX2    MLENLY-KQGIKTPTAEQIQQITGRLQAFGHIEGKNVFYWFQNHKARQRQK
AeWOX2    MLENLY-KQGIKTPTAEQIQQITGRLQAFGHIEGKNVFYWFQNHKARQRQK
AcWOX9b   ILEAIFNS-GMVNPPRDEIRKIRTQLQEYGQVGDANVFYWFQNRKSRTKHK
AeWOX9    ILEAIFNS-GMVNPPRDEIRKIRIQLQEYGQVGDANVFYWFQNRKSRTKHK
AcWOX5    VLTDLF-RSGLRTPSTDQIQKISSQLSFYGKIESKNVFYWFQNHKARERQK
AcWOX11b  ILESIFNS-GMVNPPKDETVRIRKLLEKFGAVGDANVFYWFQNRRSRSRRR
AeWOX11   ILESIFNS-GMVNPPKDETVRIRKLLEKFGAVGDANVFYWFQNRRSRSRRR
AeWOX12   ILESIFNS-GMVNPPKDETVRIRKLLEKFGAVGDANVFYWFQNRRSRSRRR
AcWOX11a  ILESIFNS-GMVNPPKDETVRIRKLLEKFGTVGDANVFYWFQNRRSRSRRR
AcWOX13b  ILERIFDQ-GNGTPSKQKIKEITSELSQHGQISETNVYNWFQNRRARSKRK
AcWOX13a  ILERIFDQ-GNGTPSKQKIKEITSELSQHGQISETNVYNWFQNRRARSKRK
AeWOX13   ILERIFDQ-GNGTPSKQKIKEITSELSQHGQISETNVYNWFQNRRARSKRK
AeWOX10   -LTSHPDLSGNGTPSKQKIKEITSELSQHGQISETNVYNWFQNRRARSKRK
```
