# Supplementary material for: In silico identification, characterization expression profile of WUSCHEL-Related Homeobox (WOX) gene family in two species of kiwifruit
Source: PeerJ. 2021 Oct 28;9:e12348. doi: 10.7717/peerj.12348 (PMC8557698; doi:10.7717/peerj.12348)
Supplement: Supplemental Information 4 [file peerj-09-12348-s004.docx]

Table S1. Characteristics of kiwifruit WOX genes.

| Species | Genes | Genome ID | Chromosome | Start site | End site | CDS Length (bp) |
| --- | --- | --- | --- | --- | --- | --- |
| *A. chinensis* | AcWUS1a | Acc05206.1 | Chr5 | 528515 | 530899 | 717 |
|  | AcWOX13a | Acc05283.1 | Chr5 | 1309215 | 1313978 | 798 |
|  | AcWOX11a | Acc08359.1 | Chr8 | 636931 | 640791 | 597 |
|  | AcWOX9a | Acc09522.1 | Chr8 | 21838668 | 21842261 | 840 |
|  | AcWOX4a | Acc11781.1 | Chr10 | 16954076 | 16957617 | 666 |
|  | AcWOX4b | Acc12050.1 | Chr11 | 1221582 | 1224373 | 783 |
|  | AcWOX11b | Acc16985.1 | Chr8 | 17910949 | 17915764 | 714 |
|  | AcWOX9b | Acc17296.1 | Chr15 | 13209517 | 13213331 | 705 |
|  | AcWOX1a | Acc18757.1 | Chr16 | 22904987 | 22908204 | 1041 |
|  | AcWOX4c | Acc23762.1 | Chr21 | 3709719 | 3711498 | 708 |
|  | AcWOX13b | Acc24349.1 | Chr21 | 14665646 | 14670884 | 882 |
|  | AcWUS1b | Acc24453.1 | Chr21 | 16765733 | 16767838 | 843 |
|  | AcWOX2 | Acc25877.1 | Chr23 | 4658835 | 4661197 | 756 |
|  | AcWOX3a | Acc25908.1 | Chr23 | 5039228 | 5041172 | 1062 |
|  | AcWOX5 | Acc27793.1 | Chr24 | 12486955 | 12487893 | 888 |
|  | AcWOX1b | Acc29332.1 | Chr26 | 3594853 | 3598689 | 645 |
|  | AcWOX3b | Acc30208.1 | Chr26 | 17037283 | 17040279 | 765 |
| *A. eriantha* | AeWOX13 | DTZ79_05g01270 | Chr05 | 1774008 | 1778803 | 1083 |
|  | AeWOX11 | DTZ79_08g00560 | Chr08 | 652744 | 654595 | 672 |
|  | AeWOX4a | DTZ79_10g10530 | Chr10 | 20909193 | 20912321 | 840 |
|  | AeWOX4b | DTZ79_11g02060 | Chr11 | 2110830 | 2120045 | 855 |
|  | AeWOX12 | DTZ79_15g08240 | Chr15 | 11193930 | 11198907 | 744 |
|  | AeWOX9 | DTZ79_15g11700 | Chr15 | 16406313 | 16410015 | 630 |
|  | AeWOX4c | DTZ79_21g04040 | Chr21 | 4223262 | 4225207 | 507 |
|  | AeWOX10 | DTZ79_21g11470 | Chr21 | 17222231 | 17227082 | 1113 |
|  | AeWUS1 | DTZ79_21g12670 | Chr21 | 18739258 | 18740990 | 519 |
|  | AeWOX2 | DTZ79_23g05220 | Chr23 | 5260166 | 5262401 | 600 |
