# Supplementary material for: In silico identification, characterization expression profile of WUSCHEL-Related Homeobox (WOX) gene family in two species of kiwifruit
Source: PeerJ. 2021 Oct 28;9:e12348. doi: 10.7717/peerj.12348 (PMC8557698; doi:10.7717/peerj.12348)
Supplement: Supplemental Information 5 [file peerj-09-12348-s005.docx]

Table S2. Ka/Ks analysis for kiwifruit WOX gene pairs.

| Gene pair | Ka | Ks | Ka/Ks |
| --- | --- | --- | --- |
| AcWOX4a/AcWOX4b | 0.063 | 0.230 | 0.275 |
| AcWOX4a/AcWOX4c | 0.187 | 0.719 | 0.260 |
| AcWOX4b/AcWOX4c | 0.163 | 0.701 | 0.232 |
| AcWOX9b/AcWOX9a | 0.065 | 0.210 | 0.310 |
| AcWUS1b/AcWUS1a | 0.026 | 0.159 | 0.165 |
| AcWOX13b/AcWOX13a | 0.031 | 0.222 | 0.138 |
| AcWOX3a/AcWOX3b | 0.159 | 0.529 | 0.301 |
| AcWOX11a/AcWOX11b | 0.045 | 0.158 | 0.286 |
| AeWOX13/AeWOX10 | 0.027 | 0.226 | 0.120 |
| AeWOX11/AeWOX12 | 0.057 | 0.165 | 0.346 |
| AeWOX4a/AeWOX4b | 0.120 | 0.351 | 0.342 |
| AeWOX4a/AeWOX4c | 0.183 | 0.687 | 0.267 |
| AeWOX4b/AeWOX4c | 0.201 | 0.788 | 0.256 |
| AeWOX13/AcWOX13b | 0.026 | 0.217 | 0.120 |
| AeWOX13/AcWOX13a | 0.005 | 0.030 | 0.151 |
| AeWOX11/AcWOX11a | 0.023 | 0.052 | 0.436 |
| AeWOX11/AcWOX11b | 0.052 | 0.187 | 0.278 |
| AeWOX4a/AcWOX4a | 0.035 | 0.109 | 0.317 |
| AeWOX4a/AcWOX4b | 0.043 | 0.194 | 0.223 |
| AeWOX4a/AcWOX4c | 0.217 | 0.830 | 0.261 |
| AeWOX4b/AcWOX4a | 0.064 | 0.264 | 0.242 |
| AeWOX4b/AcWOX4b | 0.012 | 0.089 | 0.136 |
| AeWOX4b/AcWOX4c | 0.181 | 0.809 | 0.223 |
| AeWOX9/AcWOX9b | 0.019 | 0.045 | 0.427 |
| AeWOX9/AcWOX9a | 0.077 | 0.206 | 0.371 |
| AeWOX12/AcWOX11a | 0.062 | 0.159 | 0.390 |
| AeWOX12/AcWOX11b | 0.025 | 0.107 | 0.228 |
| AeWOX4c/AcWOX4a | 0.200 | 0.678 | 0.294 |
| AeWOX4c/AcWOX4b | 0.185 | 0.662 | 0.279 |
| AeWOX4c/AcWOX4c | 0.027 | 0.086 | 0.309 |
| AeWOX10/AcWOX13b | 0.004 | 0.037 | 0.096 |
| AeWUS1/AcWUS1b | 0.056 | 0.136 | 0.412 |
| AeWOX10/AcWOX13a | 0.031 | 0.224 | 0.136 |
| AeWUS1/AcWUS1a | 0.057 | 0.263 | 0.215 |
| AeWOX2/AcWOX2 | 0.007 | 0.032 | 0.235 |
